# Supplementary material for: Alcohol Septal Ablation versus Septal Myectomy Treatment of Obstructive Hypertrophic Cardiomyopathy: A Systematic Review and Meta-Analysis
Source: J Clin Med. 2020 Sep 23;9(10):3062. doi: 10.3390/jcm9103062 (PMC7598206; doi:10.3390/jcm9103062)

Supplementary data

Table 1. Main characteristics of studies included in the study.

Study year	Study design	Country	Sample size	Key endpoints	Secondary end-points	Follow-up (months)
Qin 2001	Prospective cohort	USA Cleveland	51	PC, PD	LVOTO, IVSd LVEDD, LA	3
Firoozi 2002	Prospective cohort	UK	44	All-cause mortality SCD, CV mortality PC	NYHA, Angina LVOTO, LVEDD LA size	32.2
Jing 2004	Prospective cohort	China	54	All-cause mortality		24
Ralph-Edwards 2005	Prospective cohort	Canada	102	All-cause mortality SCD, CV mortality	LVEDD, IVSd LA size	24.3
Vural 2007	Prospective cohort	Turkey	40	All-cause mortality Reintervention PC	NYHA, Angina LVOTO, LVEDD IVSd, LA size	13.12
Ball 2011	Prospective cohort	Canada	372	All-cause mortality	IVSd, LA size	60
Sorajja 2012	Retrospective cohort	USA Mayo Clinic	354	All-cause mortality SCD, CV mortality PC, Stroke	Angina, LVOTO LVEDD, LA size	68.4
Knyshov 2013	Prospective cohort	Ukraine	42	All-cause mortality	LVOTO, NYHA	31.9

Steggerda 2014	Prospective cohort	Netherlands	263	All-cause mortality<br>SCD, CV mortality<br>Reintervention, PCI	IVSd	60
Samardhi 2014	Prospective cohort	Australia	70	All-cause mortality<br>Reintervention<br>PC, PD	NYHA, Angina<br>LVOTO, IVSd<br>LA size	43
Vriesendorp 2014	Prospective cohort	Netherlands & Belgium	574	All-cause mortality<br>SCD, CV mortality<br>PC	LVOTO, IVSd	91.2
Sedehi 2015	Prospective cohort	USA Stanford	223	All-cause mortality<br>PD	NYHA<br>LVOTO, IVSd	120
Yang 2016	Prospective cohort	China	59	All-cause mortality<br>SCD, CV mortality<br>Reintervention	Angina, LVOTO<br>LVEDD, IVSd<br>LA size	36
Finocchiaro 2016	RCTs	USA Stanford	40		LVOTO, IVSd<br>LVEDD, LA size	12
Cavigli 2018	Prospective cohort	Italy	126	All-cause mortality<br>SCD, CV mortality<br>Reintervention, PD	Angina<br>LVOTO, IVSd<br>LA size	60
Guo 2018	RCTs	China	226	All-cause mortality<br>Reintervention, PD<br>PC	Angina<br>LVOTO, IVSd<br>LA size	41.2
Hoedemakers 2019	Retrospective	Belgium	77	All-cause mortality<br>SCD, CV mortality<br>PD	LVOTO, IVSd<br>LA size	84

Nguyen 2019	Prospective cohort	USA	503	All-cause mortality PC, Reintervention	LVOTO, IVSd LA size	31.2
Kimmelstiel 2019	Prospective cohort	USA Boston	477	All-cause mortality PC, Reintervention	LVOT, IVSd	48
Shimada 2019	Prospective cohort	USA California Florida	850		PC, PP	76.8

Abbreviation: CV: cardio-vascular; SCD: sudden cardiac death; PC: peri-procedural complication; PD: pacemaker dependence; LVOTO: left ventricle outflow tract obstruction; IVSd: interventricular septal in diastole; LA: left atrium; LVEDD: left ventricle end-diastolic dimension.

Table S2. Main characteristics of patients enrolled among trials included in the study

Study (year)	Arms of treatment	Number of patients	Age (year)	Female (%)	Mean hospital Stay (days)	LVOTO mmHg	NYHA class (mean)
Qin 2001	ASA	25	63 ± 14	72	5.6 ± 2.3	64 ± 39	3.5 ± 0.5
	SM	26	48 ± 13	39	8.1 ± 3.5	62 ± 43	3.3 ± 0.5
Firoozi 2002	ASA	20	49 ± 13	40	NR	83 ± 23	2.3 ± 0.5
	SM	24	38 ± 16	46	NR	91 ± 18	2.4 ± 0.6
Jing 2004	ASA	43	36 ± 11		NR	NR	NR
	SM	11	45 ± 14		NR	NR	NR
Ralph-Edwards	ASA	54	59 ± 15	52	7 days	74 ± 36	NR
2005	SM	48	46 ± 17	38	8 days	64 ± 27	NR
Vural 2007	ASA	24	25 ± 7.3	42	4.3 ± 0.9	65 ± 60	NR
	SM	16	24 ± 6.6	44	6.5 ± 1.8	72.5 ± 20	NR

Ball 2011	ASA	85	57 ± 16	50	NR	NR	NR
	SM	287	47 ± 15	40	NR	NR	NR
Sorajja 2012	ASA	177	63 ± 13	58	NR	70 ± 40.	NR
	SM	177	62 ± 12	58	NR	67 ± 40.	NR
Knyshov 2013	ASA	14	34 ± 8.5	43	NR	97.5 ± 8.9	2.14 ± 0.53
	SM	28	29.1 ± 16.1	54	NR	113.3 ± 14.9	2 ± 0.86
Steggerda 2014	ASA	161	59 ± 14	48	5 ± 3	NR	NR
	SM	102	56 ± 16	54	9 ± 6	NR	NR
Samardhi 2014	ASA	47	57 ± 14.7	45	NR	74.0 ± 20	NR
	SM	23	47 ± 20.6	56.5	NR	75.5 ±18	NR
Vriesendorp 2014	ASA	321	58 ± 14	45	NR	102 ± 52	NR
	SM	253	52 ± 16	46	NR	92 ± 39	NR
Sedehi 2015	ASA	52	57.3 ± 12.9	44	NR	67.1 ± 26.9	2.9 9 ± 0.35
	SM	171	48.0 ± 17.1	51	NR	67.4 ± 43.4	2.74 ± 0.65
Yang 2016	ASA	22	45.5 ± 8.1	20	NR	79.7 ± 21.2	NR
	SM	37	44.6 ± 9.5	33	NR	69.0 ± 23.9	NR
Finocchiaro 2016	ASA	9	54 ± 14	45	NR	48 ± 32	NR
	SM	31	50 ± 12	42	NR	59 ± 32	NR
Cavigli 2018	ASA	55	49 ±14	58	NR	70 ± 33	NR
	SM	71	42 ± 16	38	NR	52 ± 31	NR
Guo 2018	ASA	68	52.59 ± 10.54	37	NR	70.30 ± 44.79	NR
	SM	158	49.20 ± 12.30	51	NR	74.58 ± 45.52	NR
Hoedemakers 2019	ASA	25	61.18 ± 20.8	68	6.0 ± 3	45.00 ± 20	NR
	SM	52	54.49 ± 26.1	54	3.0 ± 3	64.00 ± 30	NR
Nguyen 2019	ASA	167	65 ± 14	56	NR	65.0 ± 19	NR
	SM	334	64 ±13	54	5.8 ± 5.6	60.0 ± 17	NR

Kimmelstiel 2019	ASA	99	66.3 ± 11.9	63	6.4 ± 3.3	65.7 ± 40.7	NR
	SM	378	52.7 ± 14.7	42	NR	58.0 ± 41.8	NR
Shimada 2019	ASA	393	61 ± 15	NR	5.1 ± 2.7	NR	NR
	SM	457	61 ± 15	NR	7.8 ± 4.6	NR	NR

Abbreviations: ASA: Alcohol septal ablation; SM: septal myectomy; LVOTO: left ventricle outflow tract obstruction; NR: non reported

Table S3. Assessment of risk of bias in the included studies using Cochrane criteria for CTs

**Study (trial) year**	**Sequence generation**	**Allocation concealment**	**Blinding of participants and personnel**	**Blinding of outcome assessment**	**Incomplete outcome data**	**Selective outcome reporting**	**Other bias**
Finocchiaro 2016	L	L	U	L	L	L	L
Guo 2018	L	L	U	U	L	L	L

Legend: L: low risk of bias; H: high risk of bias; U: unclear risk of bias.

Table S4. Assessment of risk of bias in the included studies using Newcastle-Ottawa Quality Assessment Scale (NOS) for cohort studies.

Study, year	Selection 1	Selection 2	Selection 3	Selection 4	Comparability 1	Exposure 1	Exposure 2	Exposure 3	Quality
Qin 2001	a)	a)	b)	a)	a)	b)	a)	b)	Good
Firoozi 2002	a)	a)	a)	a)	b)	a)	a)	a)	Good
Jing 2004	a)	b)	b)	a)	b)	a)	b)	b)	Good
Ralph-Edwards 2005	a)	a)	a)	a)	a)	b)	a)	b)	Good
Vural 2007	b)	a)	b)	b)	a)	a)	a)	b)	Good
Ball 2011	a)	b)	b)	a)	b)	b)	a)	b)	Good
Sorajja 2012	a)	a)	a)	a)	a)	a)	a)	a)	Good
Knyshov 2013	b)	a)	b)	b)	b)	d)	b)	a)	Fair
Steggerda 2014	a)	a)	b)	b)	a)	a)	b)	a)	Good
Samardhi 2014	b)	b)	a)	b)	a)	a)	b)	b)	Fair
Vriesendorp 2014	a)	a)	b)	a)	a)	a)	b)	a)	Good

Sedehi 2015	c)	b)	b)	b)	a)	b)	a)	b)	Fair
Yang 2016	b)	a)	b)	b)	b)	b)	a)	b)	Fair
Cavigli 2018	b)	a)	b)	b)	a)	a)	b)	b)	Good
Hoedemakers 2019	a)	b)	a)	a)	b)	a)	b)	a)	Good
Nguyen 2019	b)	b)	a)	a)	a)	a)	b)	b)	Good
Kimmelstiel 2019	b)	b)	a)	a)	b)	a)	b)	b)	Good
Shimada 2019	b)	b)	a)	a)	a)	a)	b)	b)	Good

Legend: NOS: Selection- 1: a),b) one star, c), d) no star; Selection- 2: a) one star, b, c) no star; Selection- 3: a), b) one star, c), d), e) no star, Selection- 4: a) one star, b) no star; Comparability: a), b) one star, c) no star; Exposure-1: a),b) one star, c), d), e) no star; Exposure-2: a) one star, b) no star; Exposure-2**:** a),b) one star, c), d), no star.

Good quality: 3 or 4 stars in selection domain AND 1 or 2 stars in comparability domain AND 2 or 3 stars in outcome/exposure domain

Fair quality: 2 stars in selection domain AND 1 or 2 stars in comparability domain AND 2 or 3 stars in outcome/exposure domain

Poor quality: 0 or 1 star in selection domain OR 0 stars in comparability domain OR 0 or 1 stars in outcome/exposure domain

Figure S1. Flow chart of study section

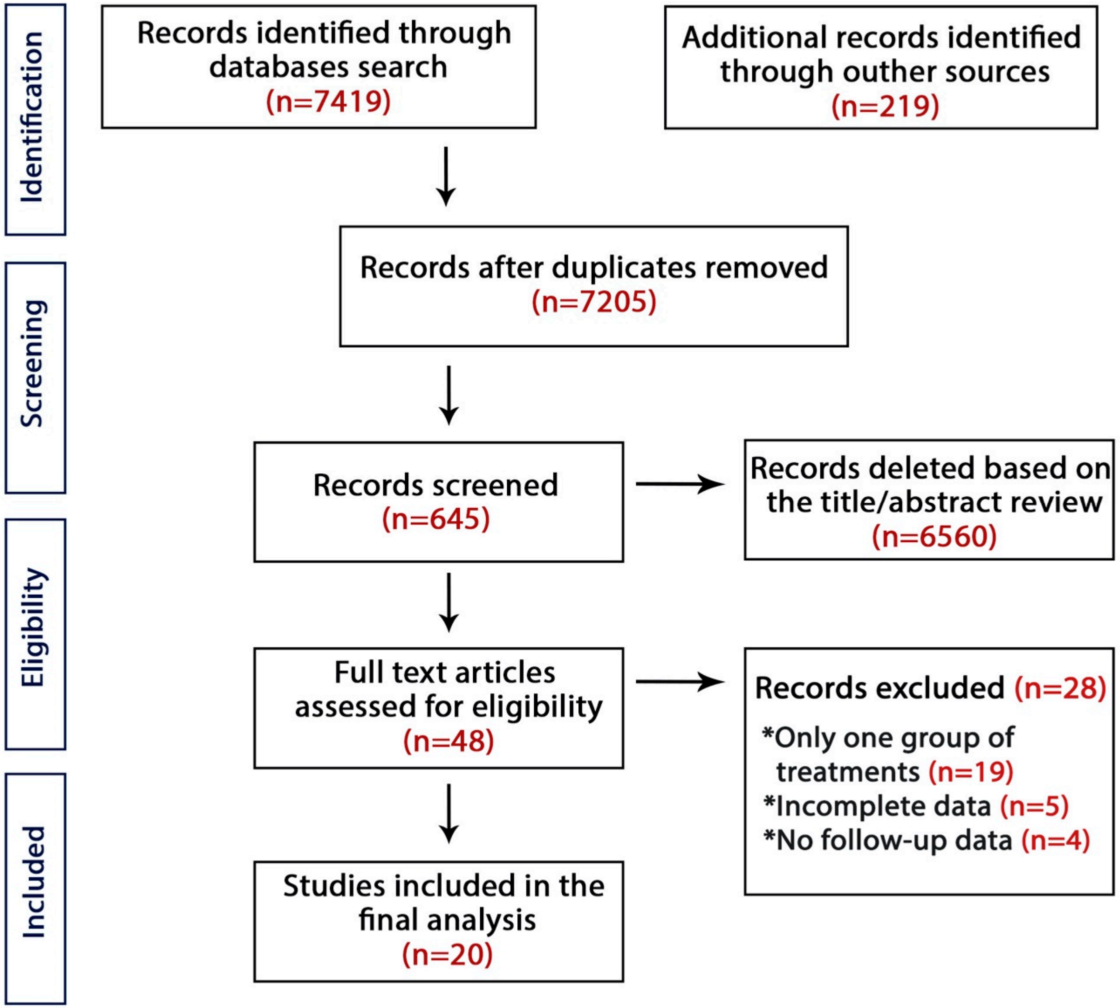

Figure S2. Baseline LV dimensions and function difference: comparison between ASA vs. SM

a) Baseline LVEDD

Study or Subgroup	ASA Mean	ASA SD	ASA Total	SM Mean	SM SD	SM Total	Weight	Mean Difference IV, Random, 95% CI	Year
Qin 2001	40	8	25	44	7	26	4.6%	−4.00 [−8.13, 0.13]	2001
Firoozi 2002	42	4	20	40	7	24	7.2%	2.00 [−1.30, 5.30]	2002
Ralph–Edwards 2005	43	5	54	43	6	48	16.8%	0.00 [−2.16, 2.16]	2005
Vural 2007	42.4	13	24	42.7	17	16	0.8%	−0.30 [−10.12, 9.52]	2007
Sorajja 2012	45	6	177	45	6	177	50.0%	0.00 [−1.25, 1.25]	2012
Yang 2016	40.1	5.9	22	39.2	4.3	37	9.8%	0.90 [−1.93, 3.73]	2016
Finocchiaro 2016	42	7	9	39	6	31	3.1%	3.00 [−2.04, 8.04]	2016
Hoedemakers 2019	40.3	5.2	20	40.4	8	52	7.9%	−0.10 [−3.25, 3.05]	2019
**Total (95% CI)**			**351**			**411**	**100.0%**	**0.13 [−0.75, 1.01]**	

Heterogeneity: $Tau^2 = 0.00$; $Chi^2 = 6.68$, df = 7 (P = 0.46); $I^2 = 0\%$
Test for overall effect: Z = 0.29 (P = 0.77)

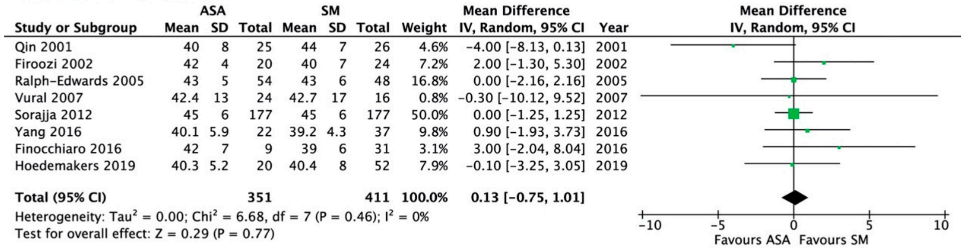

b) Baseline IVSd

Study or Subgroup	ASA Mean	ASA SD	ASA Total	SM Mean	SM SD	SM Total	Weight	Mean Difference IV, Random, 95% CI	Year
Qin 2001	23	0.4	25	24	6	26	4.2%	−1.00 [−3.31, 1.31]	2001
Ralph–Edwards 2005	22	5	54	25	6	48	4.6%	−3.00 [−5.16, −0.84]	2005
Ball 2011	19.9	4.6	85	21.8	5.3	287	9.4%	−1.90 [−3.05, −0.75]	2011
Sorajja 2012	23	5	177	22	7	177	8.7%	1.00 [−0.27, 2.27]	2012
Samardhi 2014	20.5	5.6	47	22.9	10	23	1.4%	−2.40 [−6.79, 1.99]	2014
Vriesendorp 2014	21	5	321	21	5	253	11.9%	0.00 [−0.82, 0.82]	2014
Steggerda 2014	21	9	161	20	7	102	5.4%	1.00 [−0.94, 2.94]	2014
Sedehi 2015	21.3	4.5	52	21.9	6	171	7.3%	−0.60 [−2.12, 0.92]	2015
Finocchiaro 2016	20	4	9	22	6	31	2.3%	−2.00 [−5.36, 1.36]	2016
Yang 2016	25.1	4.9	22	25.8	4.4	37	3.8%	−0.70 [−3.19, 1.79]	2016
Cavigli 2018	26	6	55	24	5	71	5.3%	2.00 [0.03, 3.97]	2018
Guo 2018	21.3	4.4	68	21.8	5.4	158	8.2%	−0.50 [−1.84, 0.84]	2018
Hoedemakers 2019	19	8	25	20.5	5.9	52	2.1%	−1.50 [−5.02, 2.02]	2019
Kimmelstiel 2019	19.6	2.9	99	20.3	4.8	378	12.4%	−0.70 [−1.45, 0.05]	2019
Nguyen 2019	19.6	2.9	167	20.3	4.8	334	13.0%	−0.70 [−1.38, −0.02]	2019
**Total (95% CI)**			**1367**			**2148**	**100.0%**	**−0.52 [−1.07, 0.04]**	

Heterogeneity: $Tau^2 = 0.49$; $Chi^2 = 28.71$, df = 14 (P = 0.01); $I^2 = 51\%$
Test for overall effect: Z = 1.83 (P = 0.07)

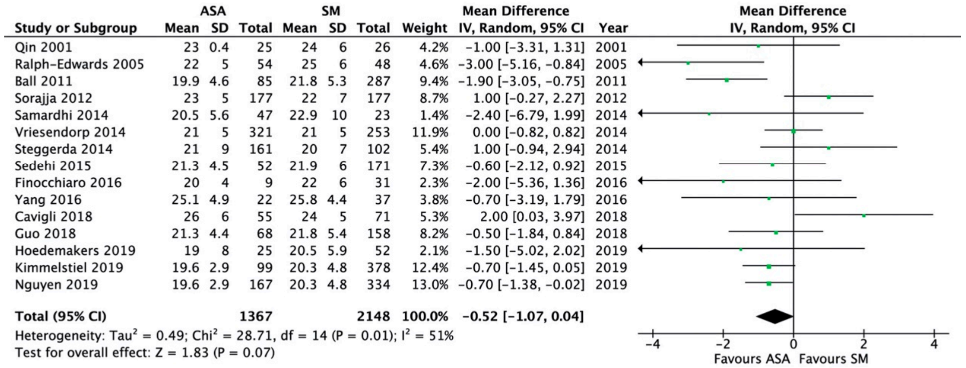

c) Baseline EF

Study or Subgroup	ASA Mean	ASA SD	ASA Total	SM Mean	SM SD	SM Total	Weight	Mean Difference IV, Random, 95% CI	Year
Qin 2001	62	5	25	63	7	26	3.5%	−1.00 [−4.33, 2.33]	2001
Samardhi 2014	70.1	6.9	47	68.8	9.4	23	2.1%	1.30 [−3.02, 5.62]	2014
Yang 2016	73	6.3	22	73.5	6.9	37	3.2%	−0.50 [−3.95, 2.95]	2016
Guo 2018	69.3	7.6	68	67.6	7.1	158	8.6%	1.70 [−0.42, 3.82]	2018
Cavigli 2018	66	10	55	67	9	71	3.4%	−1.00 [−4.37, 2.37]	2018
Hoedemakers 2019	65	8	25	65	5	52	3.3%	0.00 [−3.42, 3.42]	2019
Nguyen 2019	65	5	167	65	5	334	44.6%	0.00 [−0.93, 0.93]	2019
Kimmelstiel 2019	65	5	99	65	5	378	31.4%	0.00 [−1.11, 1.11]	2019
**Total (95% CI)**			**508**			**1079**	**100.0%**	**0.09 [−0.53, 0.71]**	

Heterogeneity: $Tau^2 = 0.00$; $Chi^2 = 3.51$, df = 7 (P = 0.83); $I^2 = 0\%$
Test for overall effect: Z = 0.28 (P = 0.78)

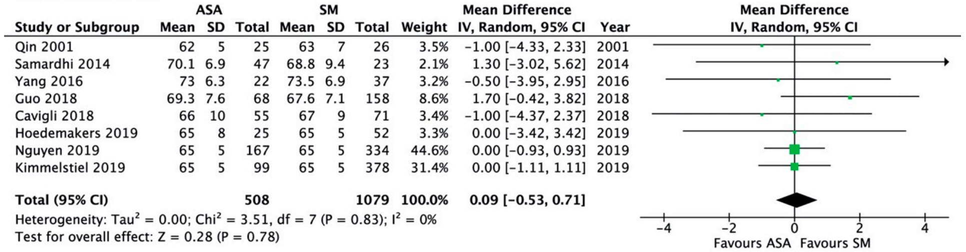

LVEDD: left ventricle end-diastolic dimension; IVSd: interventricular septum in diastole; EF: ejection fraction

Figure S3. Baseline LVOTO, MR & LA size: comparison between ASA vs. SM.

a) Baseline LVOTO

Study or Subgroup	ASA Mean	ASA SD	ASA Total	SM Mean	SM SD	SM Total	Weight	Mean Difference IV, Random, 95% CI	Year
Qin 2001	64	39	25	62	43	26	2.8%	2.00 [−20.51, 24.51]	2001
Firoozi 2002	83	23	20	91	18	24	6.1%	−8.00 [−20.39, 4.39]	2002
Ralph-Edwards 2005	74	36	54	64	27	48	6.1%	10.00 [−2.27, 22.27]	2005
Vural 2007	65	60	24	72.5	20	16	2.3%	−7.50 [−33.43, 18.43]	2007
Ball 2011	63	31	246	70	33	403	10.5%	−7.00 [−12.04, −1.96]	2011
Sorajja 2012	70	40	177	67	40	177	8.4%	3.00 [−5.33, 11.33]	2012
Samardhi 2014	74	59	47	75	38	23	2.7%	−1.00 [−23.93, 21.93]	2014
Vriesendorp 2014	102	52	321	92	39	253	9.0%	10.00 [2.55, 17.45]	2014
Sedehi 2015	67.1	26.9	52	67.4	43.4	171	7.5%	−0.30 [−10.09, 9.49]	2015
Finocchiaro 2016	48	32	9	59	32	31	2.6%	−11.00 [−34.75, 12.75]	2016
Yang 2016	79.7	21	33	69	23.9	37	7.1%	10.70 [0.18, 21.22]	2016
Cavigli 2018	70	33	55	52	31	71	6.6%	18.00 [6.68, 29.32]	2018
Guo 2018	70	45	68	74	45	158	5.9%	−4.00 [−16.79, 8.79]	2018
Hoedemakers 2019	45	41	25	64	47	52	3.2%	−19.00 [−39.53, 1.53]	2019
Kimmelstiel 2019	65.7	41	99	58	41	378	7.9%	7.70 [−1.37, 16.77]	2019
Nguyen 2019	65	20	167	60	19	334	11.3%	5.00 [1.35, 8.65]	2019
**Total (95% CI)**			**1422**			**2202**	**100.0%**	**2.37 [−1.94, 6.68]**	

Heterogeneity: Tau² = 39.46; Chi² = 41.77, df = 15 (P = 0.0002); I² = 64%
Test for overall effect: Z = 1.08 (P = 0.28)

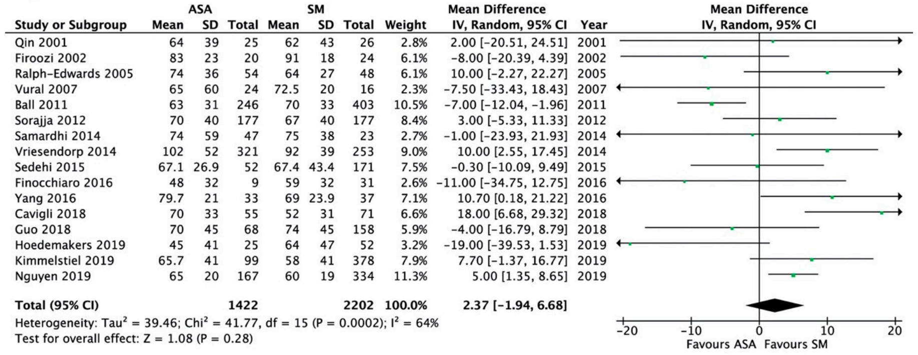

b) Baseline MR

Study or Subgroup	ASA Events	ASA Total	SM Events	SM Total	Weight	Risk Ratio M-H, Random, 95% CI	Year
Ralph-Edwards 2005	16	54	7	48	9.2%	2.03 [0.91, 4.52]	2005
Finocchiaro 2016	3	9	11	31	5.9%	0.94 [0.33, 2.65]	2016
Cavigli 2018	7	55	15	71	8.7%	0.60 [0.26, 1.37]	2018
Guo 2018	37	68	114	158	36.7%	0.75 [0.59, 0.96]	2018
Nguyen 2019	74	167	153	334	39.6%	0.97 [0.79, 1.19]	2019
**Total (95% CI)**		**353**		**642**	**100.0%**	**0.91 [0.69, 1.18]**	
Total events	137		300				

Heterogeneity: Tau² = 0.04; Chi² = 7.56, df = 4 (P = 0.11); I² = 47%
Test for overall effect: Z = 0.73 (P = 0.47)

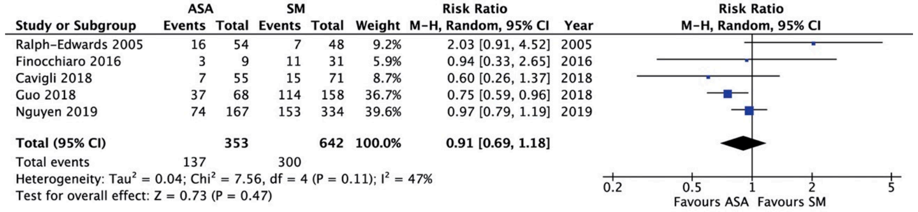

c) Baseline LA size

Study or Subgroup	ASA Mean	ASA SD	ASA Total	SM Mean	SM SD	SM Total	Weight	Mean Difference IV, Random, 95% CI	Year
Qin 2001	47	8	25	50	7	26	4.9%	−3.00 [−7.13, 1.13]	2001
Firoozi 2002	47	11	20	48	8	24	2.5%	−1.00 [−6.79, 4.79]	2002
Ralph-Edwards 2005	47	6	54	47	8	48	10.8%	0.00 [−2.77, 2.77]	2005
Ball 2011	44.4	7.3	85	45.9	7.9	285	25.6%	−1.50 [−3.30, 0.30]	2011
Yang 2016	41.8	5.5	22	42	6.6	37	8.5%	−0.20 [−3.33, 2.93]	2016
Cavigli 2018	51	7	55	50	10	71	9.4%	1.00 [−1.97, 3.97]	2018
Guo 2018	41.2	5.5	68	42.9	6.7	158	29.7%	−1.70 [−3.37, −0.03]	2018
Hoedemakers 2019	41.5	6.5	25	45.1	6.7	52	8.5%	−3.60 [−6.73, −0.47]	2019
**Total (95% CI)**			**354**			**701**	**100.0%**	**−1.29 [−2.20, −0.38]**	

Heterogeneity: Tau² = 0.00; Chi² = 6.62, df = 7 (P = 0.47); I² = 0%
Test for overall effect: Z = 2.77 (P = 0.006)

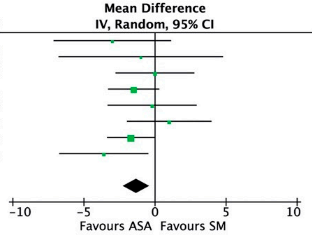

LVOTO: left ventricle outflow tract obstruction; MR: mitral regurgitation; LA: left atrium

Figure S4. NYHA class mean change between ASA vs. SM

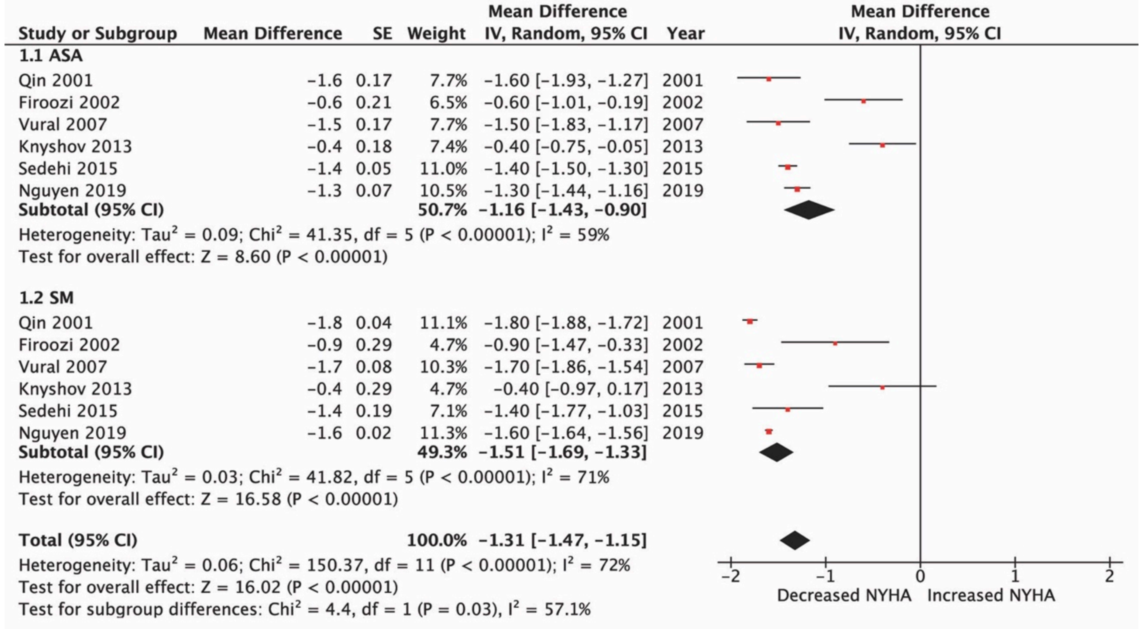

Figure S5. LV dimensions mean change; a) LVEDD; b) IVSd

a) LVEDD mean change; a) ASA group; b) SM group

Study or Subgroup	Mean Difference	SE	Weight	Mean Difference IV, Random, 95% CI	Year
**1.19.1 ASA**					
Qin 2001	4	2.26	3.0%	4.00 [−0.43, 8.43]	2001
Firoozi 2002	2	1.61	5.7%	2.00 [−1.16, 5.16]	2002
Vural 2007	3.7	0.53	30.7%	3.70 [2.66, 4.74]	2007
Yang 2016	1.2	1.95	4.0%	1.20 [−2.62, 5.02]	2016
Hoedemakers 2019	5.5	1.33	8.0%	5.50 [2.89, 8.11]	2019
**Subtotal (95% CI)**			**51.4%**	**3.61 [2.51, 4.72]**	
Heterogeneity: Tau² = 0.26; Chi² = 4.60, df = 4 (P = 0.33); I² = 13%					
Test for overall effect: Z = 6.39 (P < 0.00001)					
**1.19.2 SM**					
Qin 2001	1	1.81	4.6%	1.00 [−2.55, 4.55]	2001
Firoozi 2002	1	1.81	4.6%	1.00 [−2.55, 4.55]	2002
Vural 2007	3.1	0.67	23.2%	3.10 [1.79, 4.41]	2007
Yang 2016	2.3	1.18	9.8%	2.30 [−0.01, 4.61]	2016
Hoedemakers 2019	5.1	1.51	6.4%	5.10 [2.14, 8.06]	2019
**Subtotal (95% CI)**			**48.6%**	**2.80 [1.66, 3.94]**	
Heterogeneity: Tau² = 0.26; Chi² = 4.67, df = 4 (P = 0.32); I² = 14%					
Test for overall effect: Z = 4.83 (P < 0.00001)					
**Total (95% CI)**			**100.0%**	**3.22 [2.43, 4.01]**	
Heterogeneity: Tau² = 0.24; Chi² = 10.67, df = 9 (P = 0.30); I² = 16%					
Test for overall effect: Z = 8.02 (P < 0.00001)					
Test for subgroup differences: Chi² = 1.00, df = 1 (P = 0.32), I² = 0.4%					

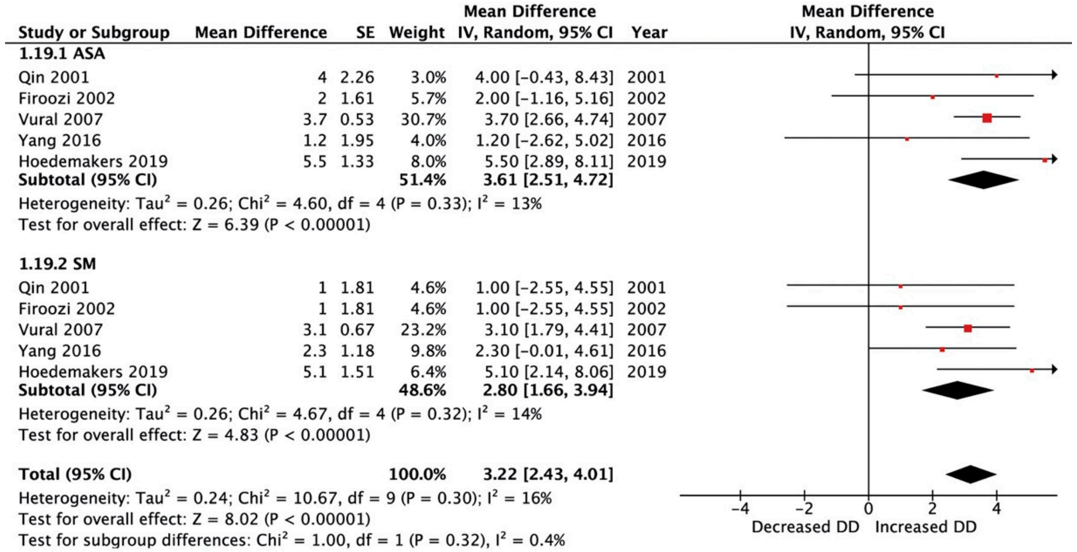

b) IVSd mean change; a) ASA group; b) SM group

Study or Subgroup	Mean Difference	SE	Weight	Mean Difference IV, Random, 95% CI	Year
**1.17.1 ASA**					
Qin 2001	−4	0.11	19.2%	−4.00 [−4.22, −3.78]	2001
Samardhi 2014	−4.6	1.21	4.5%	−4.60 [−6.97, −2.23]	2014
Sedehi 2015	−5.8	0.75	8.5%	−5.80 [−7.27, −4.33]	2015
Yang 2016	−3	1.33	3.9%	−3.00 [−5.61, −0.39]	2016
Cavigli 2018	−4	1.05	5.5%	−4.00 [−6.06, −1.94]	2018
Guo 2018	−2.6	0.72	8.9%	−2.60 [−4.01, −1.19]	2018
Hoedemakers 2019	−5	1.78	2.4%	−5.00 [−8.49, −1.51]	2019
**Subtotal (95% CI)**			**52.9%**	**−4.07 [−4.84, −3.31]**	
Heterogeneity: Tau² = 0.39; Chi² = 10.66, df = 6 (P = 0.10); I² = 44%					
Test for overall effect: Z = 10.43 (P < 0.00001)					
**1.17.2 SM**					
Qin 2001	−7	1.24	4.3%	−7.00 [−9.43, −4.57]	2001
Samardhi 2014	−4.3	2.67	1.1%	−4.30 [−9.53, 0.93]	2014
Sedehi 2015	−4	0.68	9.5%	−4.00 [−5.33, −2.67]	2015
Yang 2016	−5.8	1.05	5.5%	−5.80 [−7.86, −3.74]	2016
Cavigli 2018	−4	0.83	7.6%	−4.00 [−5.63, −2.37]	2018
Guo 2018	−5.3	0.53	11.9%	−5.30 [−6.34, −4.26]	2018
Hoedemakers 2019	−5	0.87	7.1%	−5.00 [−6.71, −3.29]	2019
**Subtotal (95% CI)**			**47.1%**	**−4.95 [−5.65, −4.25]**	
Heterogeneity: Tau² = 0.14; Chi² = 7.15, df = 6 (P = 0.31); I² = 16%					
Test for overall effect: Z = 13.86 (P < 0.00001)					
**Total (95% CI)**			**100.0%**	**−4.50 [−5.07, −3.93]**	
Heterogeneity: Tau² = 0.43; Chi² = 25.75, df = 13 (P = 0.02); I² = 50%					
Test for overall effect: Z = 15.46 (P < 0.00001)					
Test for subgroup differences: Chi² = 2.72, df = 1 (P = 0.10), I² = 63.3%					

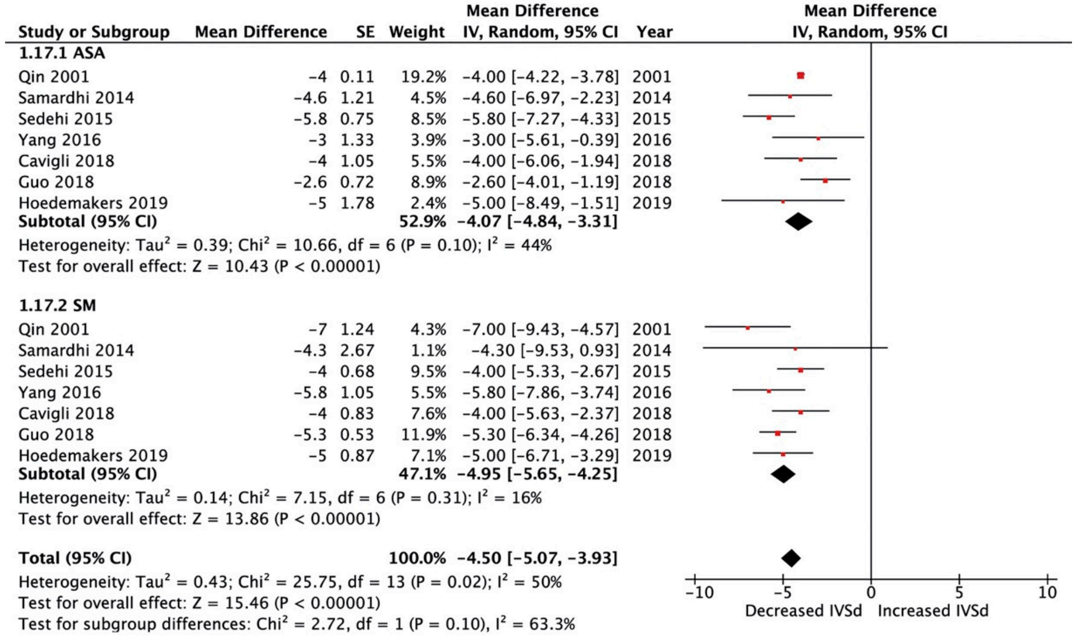

Figure S6. LV systolic function and LA size mean change; a) LVEF; b) LA size

a) LVEF mean change; a) ASA group; b) SM group

Study or Subgroup	Mean Difference	SE	Weight	Mean Difference IV, Random, 95% CI	Year
**1.21.1 ASA**					
Qin 2001	−2	1.56	9.8%	−2.00 [−5.06, 1.06]	2001
Samardhi 2014	−3.9	1.56	9.8%	−3.90 [−6.96, −0.84]	2014
Yang 2016	−4	2.17	6.0%	−4.00 [−8.25, 0.25]	2016
Cavigli 2018	−5	2.01	6.7%	−5.00 [−8.94, −1.06]	2018
Guo 2018	−5.2	1.12	14.6%	−5.20 [−7.40, −3.00]	2018
**Subtotal (95% CI)**			**46.9%**	**−4.16 [−5.52, −2.80]**	
Heterogeneity: Tau² = 0.00; Chi² = 2.99, df = 4 (P = 0.56); I² = 0%					
Test for overall effect: Z = 6.00 (P < 0.00001)					
**1.21.2 SM**					
Qin 2001	−5	1.65	9.0%	−5.00 [−8.23, −1.77]	2001
Samardhi 2014	−4.1	2.86	3.7%	−4.10 [−9.71, 1.51]	2014
Yang 2016	−3.5	1.66	9.0%	−3.50 [−6.75, −0.25]	2016
Cavigli 2018	−2	1.35	11.8%	−2.00 [−4.65, 0.65]	2018
Guo 2018	−6.6	0.81	19.6%	−6.60 [−8.19, −5.01]	2018
**Subtotal (95% CI)**			**53.1%**	**−4.43 [−6.47, −2.39]**	
Heterogeneity: Tau² = 2.98; Chi² = 9.75, df = 4 (P = 0.04); I² = 59%					
Test for overall effect: Z = 4.26 (P < 0.0001)					
**Total (95% CI)**			**100.0%**	**−4.36 [−5.51, −3.21]**	
Heterogeneity: Tau² = 1.11; Chi² = 13.68, df = 9 (P = 0.13); I² = 34%					
Test for overall effect: Z = 7.41 (P < 0.00001)					
Test for subgroup differences: Chi² = 0.04, df = 1 (P = 0.83), I² = 0%					

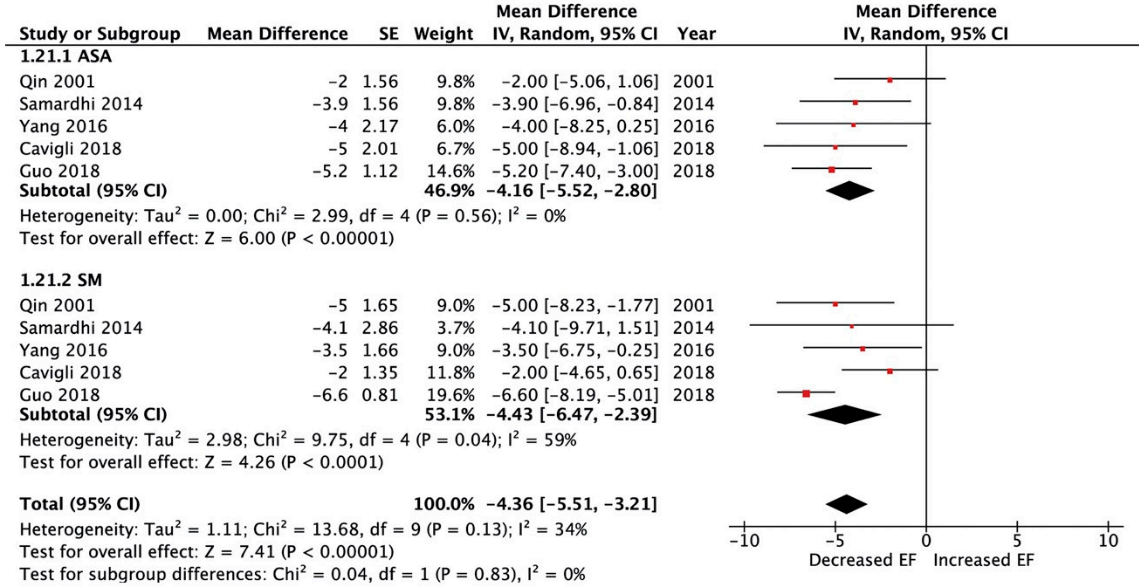

b) LA size mean change; a) ASA group; b) SM group

Study or Subgroup	Mean Difference	SE	Weight	Mean Difference IV, Random, 95% CI	Year
**1.23.1 ASA**					
Qin 2001	−1	2.26	6.3%	−1.00 [−5.43, 3.43]	2001
Firoozi 2002	−3	1.07	12.5%	−3.00 [−5.10, −0.90]	2002
Yang 2016	−1.4	1.96	7.5%	−1.40 [−5.24, 2.44]	2016
Guo 2018	−1.4	1.08	12.4%	−1.40 [−3.52, 0.72]	2018
Cavigli 2018	−2	1.33	10.8%	−2.00 [−4.61, 0.61]	2018
**Subtotal (95% CI)**			**49.6%**	**−2.00 [−3.18, −0.82]**	
Heterogeneity: Tau² = 0.00; Chi² = 1.47, df = 4 (P = 0.83); I² = 0%					
Test for overall effect: Z = 3.32 (P = 0.0009)					
**1.23.2 SM**					
Qin 2001	−3	1.94	7.6%	−3.00 [−6.80, 0.80]	2001
Firoozi 2002	−3	2.31	6.2%	−3.00 [−7.53, 1.53]	2002
Yang 2016	−2.3	1.43	10.2%	−2.30 [−5.10, 0.50]	2016
Guo 2018	−5.6	0.44	16.3%	−5.60 [−6.46, −4.74]	2018
Cavigli 2018	−4	1.44	10.2%	−4.00 [−6.82, −1.18]	2018
**Subtotal (95% CI)**			**50.4%**	**−4.09 [−5.69, −2.48]**	
Heterogeneity: Tau² = 1.51; Chi² = 7.70, df = 4 (P = 0.10); I² = 48%					
Test for overall effect: Z = 4.98 (P < 0.00001)					
**Total (95% CI)**			**100.0%**	**−2.90 [−4.30, −1.50]**	
Heterogeneity: Tau² = 2.94; Chi² = 27.31, df = 9 (P = 0.001); I² = 60%					
Test for overall effect: Z = 4.06 (P < 0.0001)					
Test for subgroup differences: Chi² = 4.18, df = 1 (P = 0.04), I² = 56.1%					

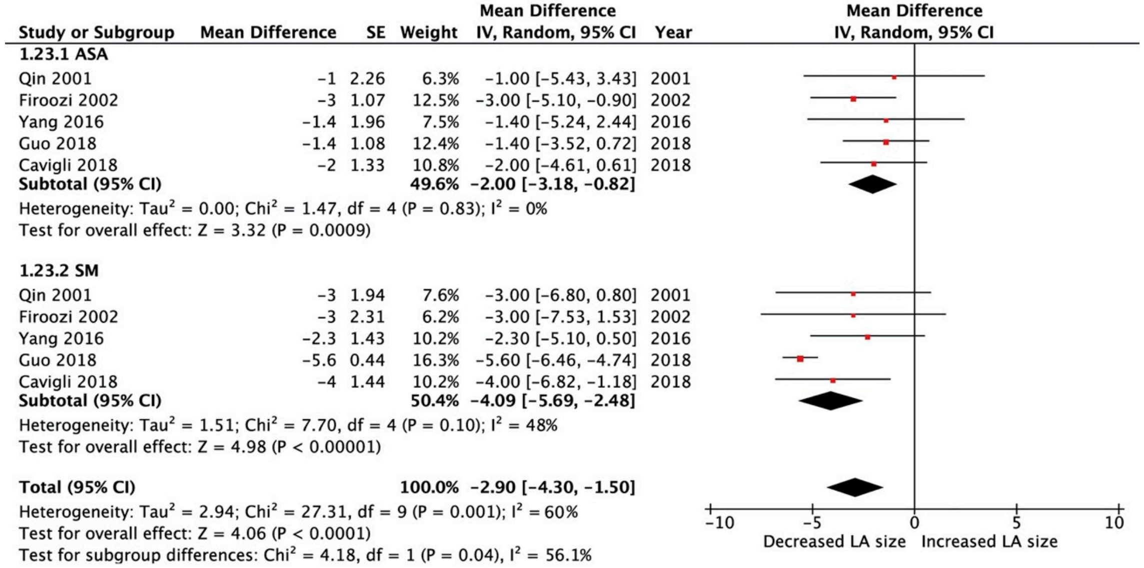

Figure S7. Meta-regression for interaction of age, female gender, LVOT and IVSd with peri-procedural complications; a) Age; b) Female; c) LVOT mean reduction; d) IVSd mean reduction.

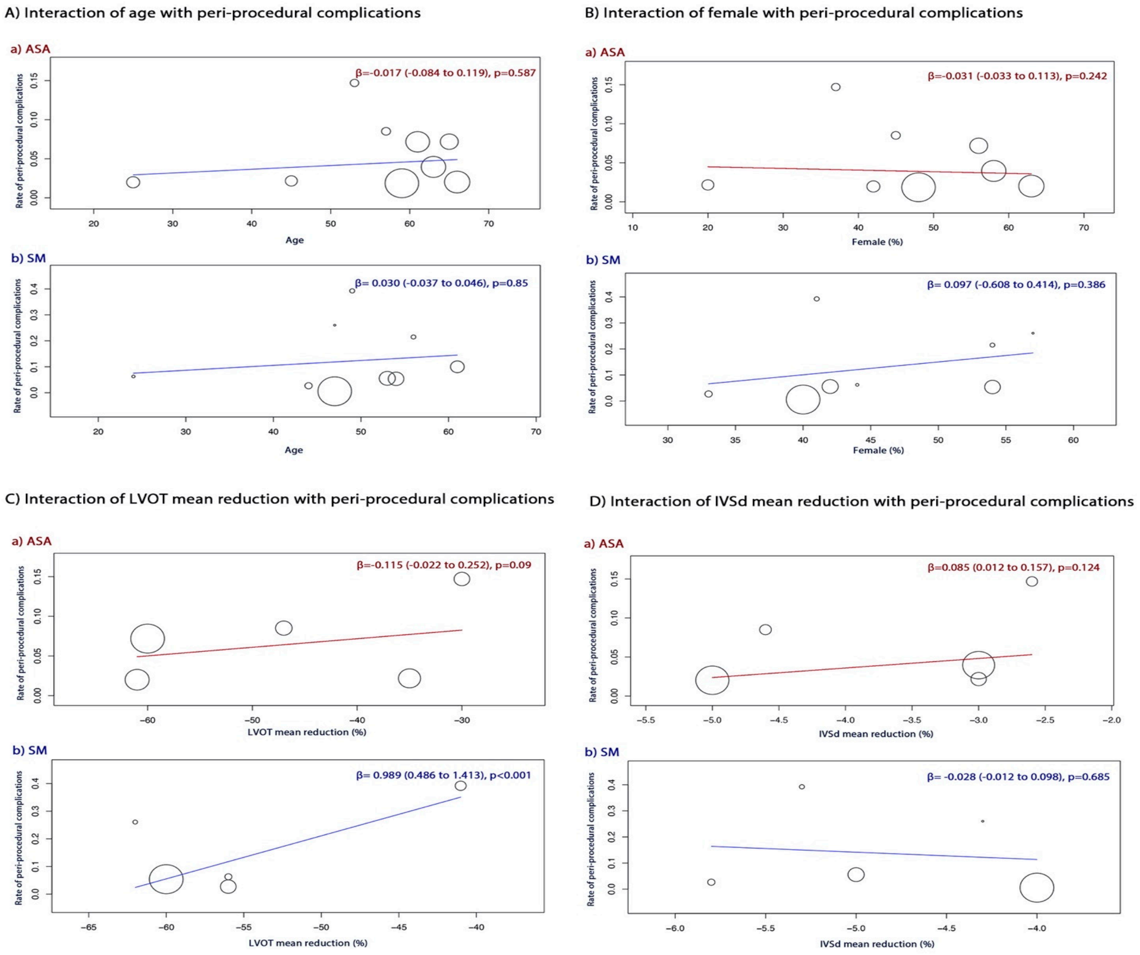

Figure S8. Meta-regression for interaction of year of publication, follow-up, age and female with all-cause mortality; a) Year of publication; b) follow-up; c) age; d) Female gender

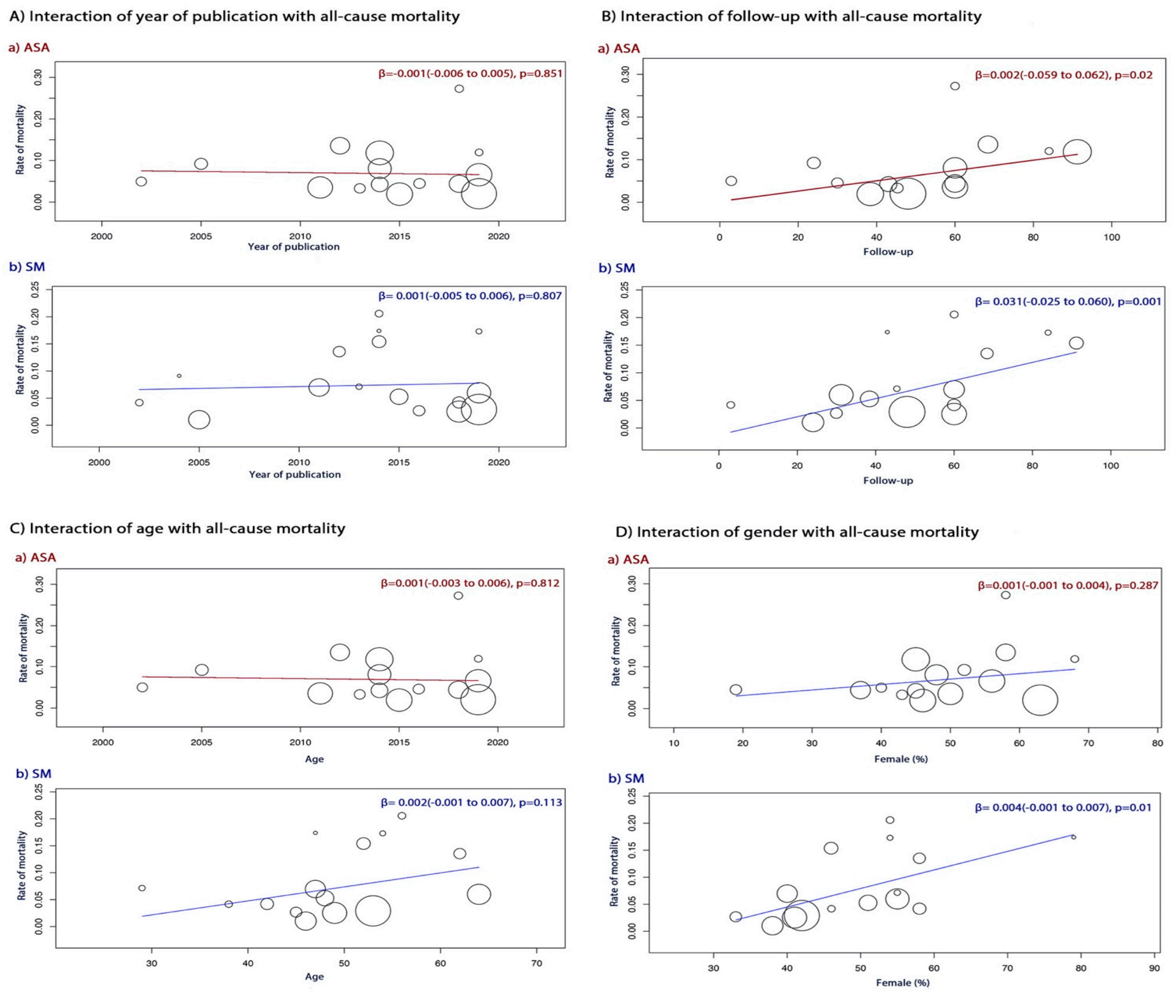

Figure S9. Meta-regression for interaction of LVOT, IVSd and LA size with all-cause mortality; a) LVOT; b) IVSd; c) LA size;

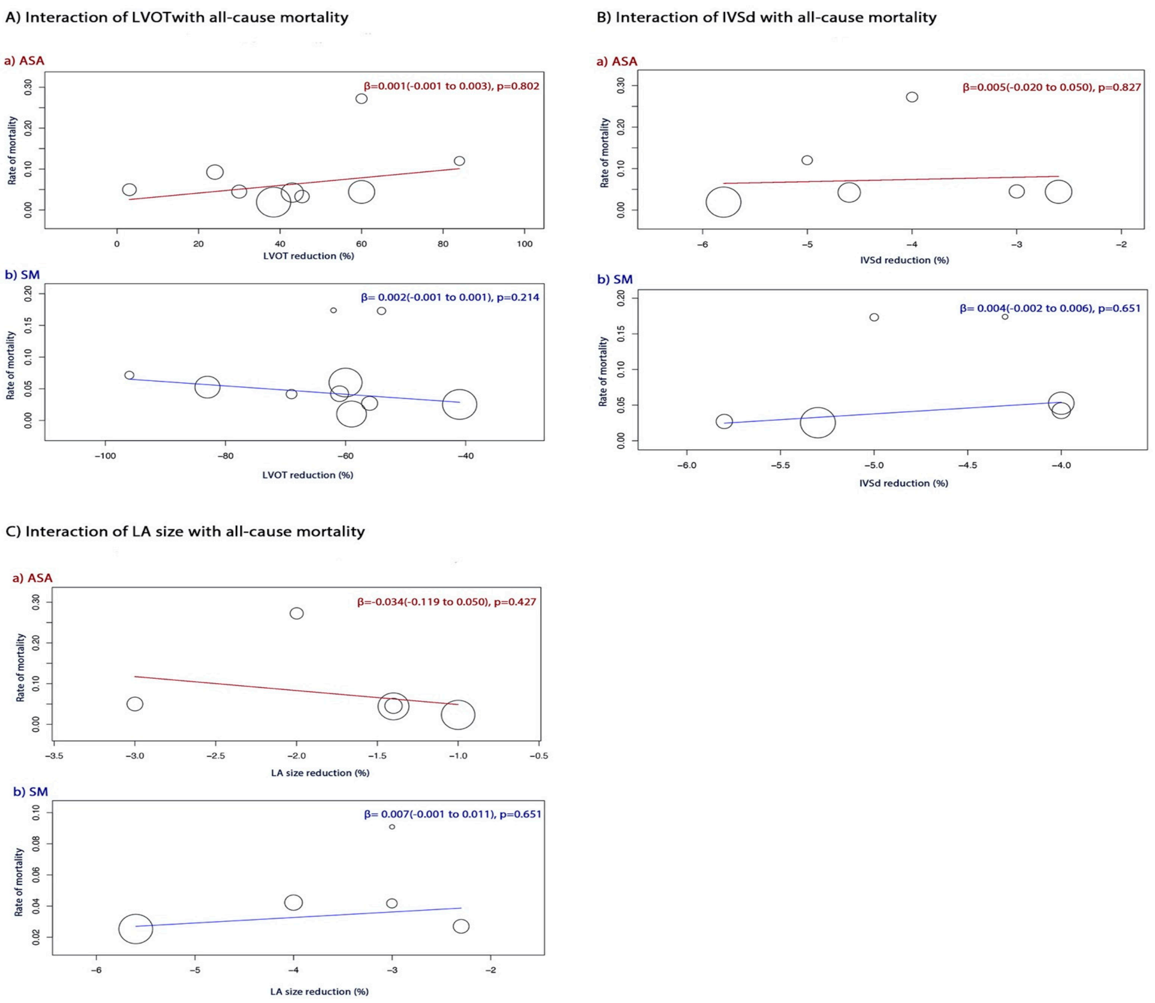

Figure S10. Meta-regression for interaction of age, female gender, LVOT and IVSd with short-term mortality; a) Age; b) Female; c) LVOT mean reduction; d) IVSd mean reduction.

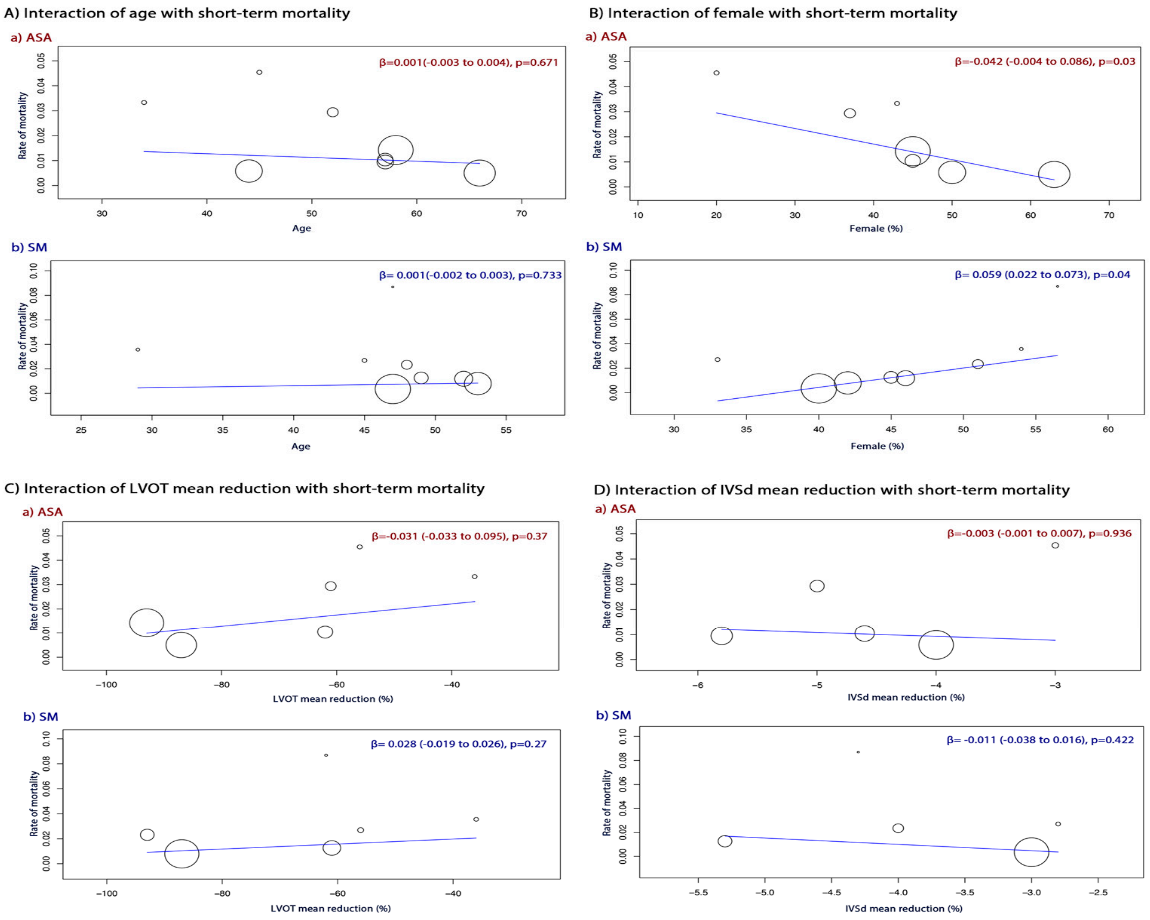

Figure S11. Meta-regression for interaction of age, female gender, LVOT and IVSd with NYHA class; a) Age; b) Female; c) LVOT mean reduction; d) IVSd mean reduction.

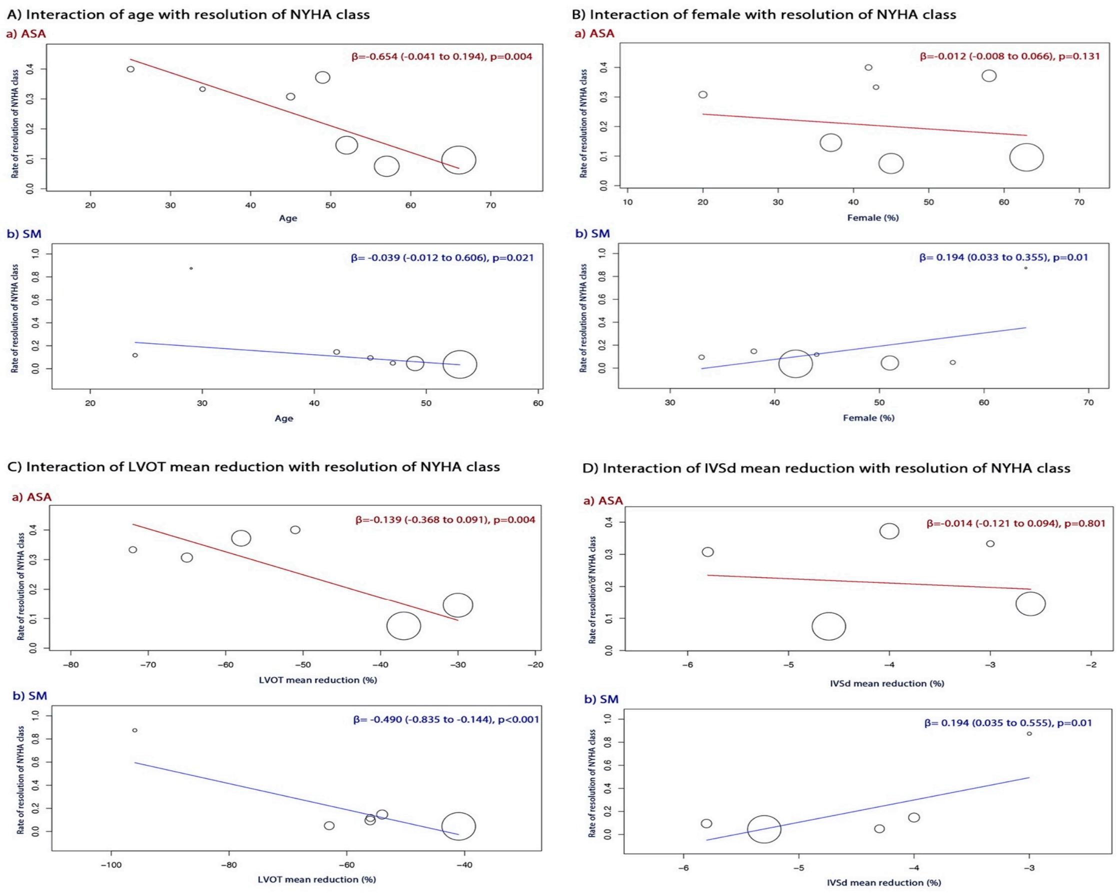

Figure S12. Meta-regression for interaction of year of publication with clinical symptoms A) NYHA class; B) Angina

A) Interaction of year of publication with resolution of NYHA class

a) ASA

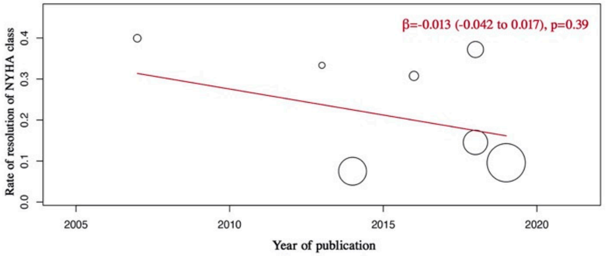

b) SM

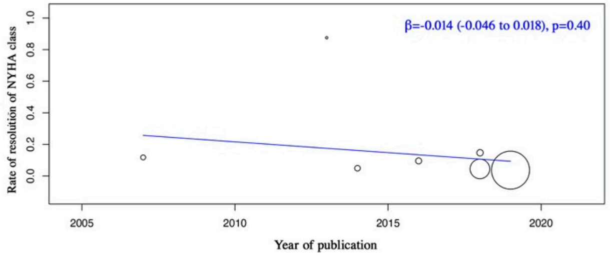

B) Interaction of year of publication with resolution of angina

a) ASA

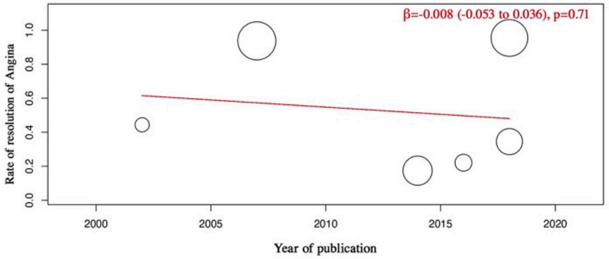

b) SM

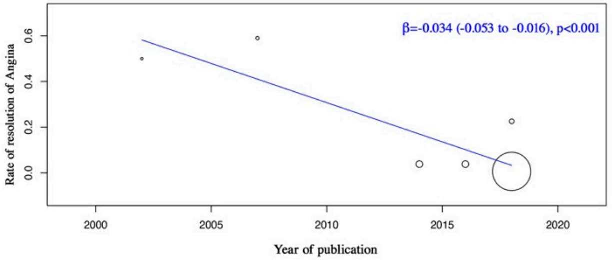

Figure S13. Meta-regression for interaction of age, female gender, LVOT and IVSd with Angina; a) Age; b) Female; c) LVOT mean reduction; d) IVSd mean reduction.

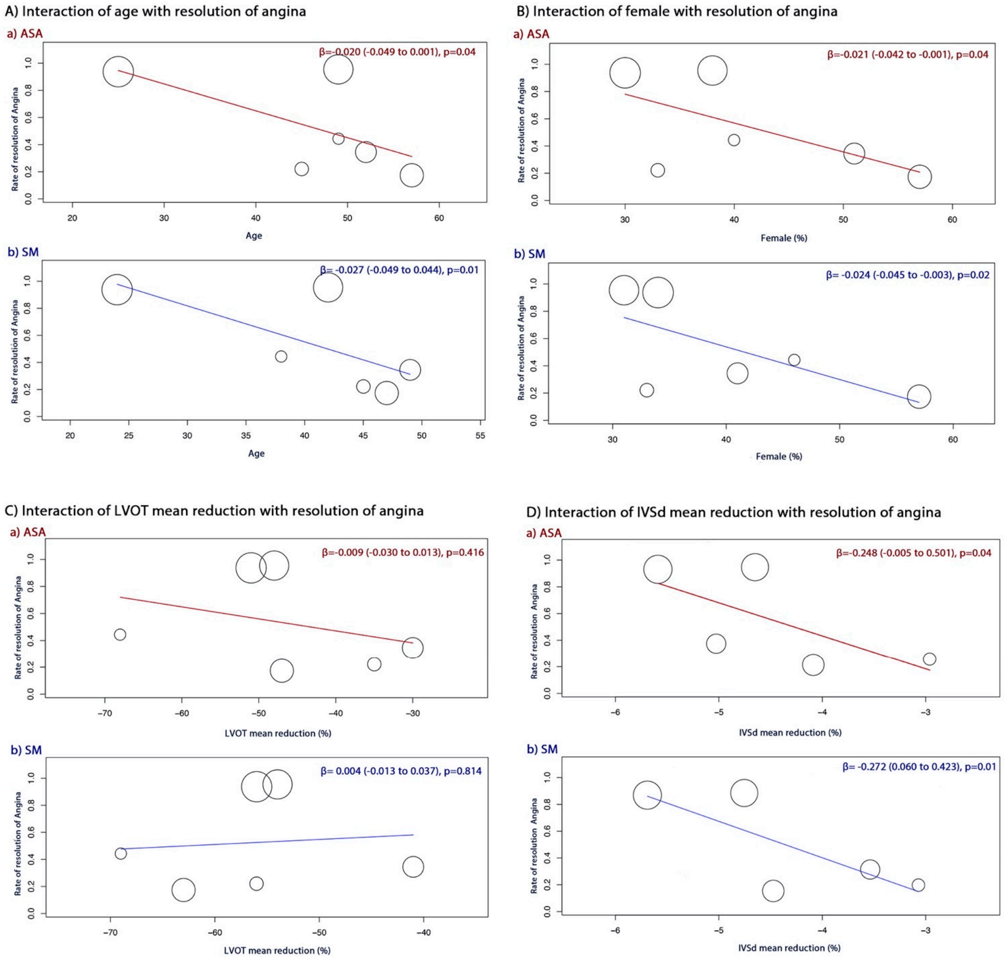

Supplement: Supplementary file 1 [file jcm-09-03062-s001.pdf]
